# Association Between Diet Quality, Physical Activity, and the Risk of Aortic Dissection—A Prospective Cohort Study

**DOI:** 10.3390/jcdd13030142

**Published:** 2026-03-18

**Authors:** Zahra Parvan, Yasmin Soltanzadeh-Naderi, Stefan Acosta

**Affiliations:** 1 Department of Clinical Sciences, Lund University, 214 21 Malmö, Sweden; za1317pa-s@student.lu.se (Z.P.); yasmin.soltanzadeh-naderi@med.lu.se (Y.S.-N.); 2 Vascular Center, Department of Cardiothoracic and Vascular Surgery, Skane University Hospital, 205 02 Malmö, Sweden

**Keywords:** aortic dissection, lifestyle factors, diet quality, physical activity, Malmö diet and cancer study

## Abstract

Background: Hypertension and smoking are well-known risk factors for aortic dissection (AD), but it is unclear whether other lifestyle factors influence the risk. The aim of this study was to investigate the association between diet quality, physical activity, and risk of AD. Methods: The study included 28,094 participants from the Malmö Diet and Cancer Study, recruited between 1991 and 1996 and followed through national health registers until 31 December 2022. Diet quality was assessed using a six-component Diet Quality Index. Incident AD was identified through ICD-9/ICD-10 codes in the Swedish National Patient Register. Associations were analyzed using Cox proportional hazards models. Results: During a median follow-up of 26.6 years, 130 participants developed AD. Higher age (HR 1.76/one standard deviation increment, 95% CI 1.41–2.20), male sex (HR 1.58, 95% CI 1.06–2.34), hypertension (HR 1.57, 95% CI 1.01–2.44), and current smoking (HR 3.04, 95% CI 1.86–4.98) were each independently associated with increased AD risk. No significant associations were found for diet quality and physical activity with AD risk. Conclusions: No associations were found between diet quality and physical activity and AD risk. Blood pressure control and smoking cessation are key preventive measures to reduce incidence of AD.

## 1. Introduction

Aortic dissection (AD) occurs when a tear develops in the intimal layer, allowing blood to enter the media under systemic pressure. This results in separation of the medial lamellae and formation of a dissection flap, which divides the aortic lumen into a true and a false lumen. The dissection may propagate in either an antegrade or retrograde direction along the aorta, potentially compromising branch vessels and causing malperfusion syndromes, in which end-organ ischemia arises from reduced perfusion of aortic branch vessels. Major complications include acute aortic regurgitation, myocardial ischemia, pericardial tamponade, stroke, and rupture. In some cases, blood within the false lumen may re-enter the true lumen through a secondary tear, but if it instead breaches the adventitia, aortic rupture ensues, an event that is almost universally fatal [1,2,3].

The wall of the aorta, like that of other large arteries, consists of three layers: the tunica intima, a thin inner layer lined by endothelium and supported by a delicate connective tissue matrix; the tunica media, a thick middle layer characterized by concentric sheets of elastic and collagen fibers interspersed with smooth muscle cells, providing the vessel with its tensile strength and elasticity; and the tunica adventitia, an outer layer composed mainly of collagen fibers, vasa vasorum, and lymphatics that nourish and stabilize the aortic wall [1,2].

Degeneration of the tunica media, often involving fragmentation of elastic fibers, loss of smooth muscle cells, and accumulation of proteoglycans, plays a central role in the development of AD. These structural changes weaken the aortic wall, making it more susceptible to intimal tearing under increased hemodynamic stress [4].

Advanced age and male sex are consistently associated with higher incidence of AD [5]. Heritable connective tissue disorders, such as Marfan, Loeys–Dietz, and vascular Ehlers–Danlos syndromes, predispose to earlier onset [6], while congenital conditions including bicuspid aortic valve and thoracic aortic aneurysm further increase risk [3,5,7]. Early recognition and imaging surveillance in patients and their first-degree relatives with heritable thoracic aortic disease are therefore essential for prevention [1].

Arterial hypertension remains the most significant determinant and is present in most patients with AD [3,7,8]. Smoking is another established risk factor; population-based studies have shown that current smokers have more than twice the risk of AD compared with never-smokers [9], whereas cessation markedly reduces risk [10]. Prospective cohort data from the Malmö Diet and Cancer Study (MDCS) indicate that smoking and dyslipidemia are more strongly associated with aneurysmal disease, whereas hypertension is the dominant risk factor for AD [8]. Findings from a Danish nationwide cohort further support these patterns, showing that hypertension, age and male sex were the most prevalent and consistent risk factors across both type A and type B dissections, with mortality remaining high despite modern management [5].

Despite these insights, little is known about the role of lifestyle factors in the development of AD. In contrast to atherosclerotic cardiovascular disease, where diet quality and physical activity are well established as major determinants of risk [11,12,13,14], no large-scale prospective cohort study has investigated their influence on AD. Findings from a large population-based cohort suggest a potential protective effect of micronutrients such as vitamin C and vitamin E, but these analyses focused on single nutrients rather than overall dietary patterns [15]. The DASH (Dietary Approaches to Stop Hypertension) dietary pattern, emphasizing the consumption of fruits, vegetables, whole grains, legumes, nuts, lean protein, and low-fat dairy products, as well as limiting intakes of salt, added sugar, and saturated fat, has been shown to reduce the risk of hypertension [16]. Similarly, both weekend warrior and regular physical activity patterns have been shown to be associated with reduced risk of hypertension [17]. A first exploratory step would be to investigate whether dietary components and physical activity, independently from hypertension, are associated with AD risk. Thus, the influence of broader lifestyle factors on AD risk remains largely unexplored.

The MDCS is a large population-based prospective cohort initiated in Malmö, Sweden, in the early 1990s. The MDCS provides comprehensive information on diet, lifestyle, and long-term health outcomes, offering an opportunity to explore the association between lifestyle factors and AD [18]. The aim of this study is therefore to explore the association between diet quality, physical activity, and risk of AD.

## 2. Material and Methods

### 2.1. Study Design and Population

This study is based on data from the MDCS cohort, a population-based prospective cohort conducted in Malmö, Sweden, between 1991 and 1996 as part of the European Prospective Investigation into Cancer and Nutrition (EPIC). The MDCS was established in the early 1990s to examine the relationship between diet, nutrition, lifestyle, and the risk of cancer [18,19]. Over time, it has also been utilized to investigate diet and lifestyle in a wide range of other chronic diseases within the EPIC framework, including cardiovascular disease, diabetes, and metabolic disorders [18].

**Figure 1:**
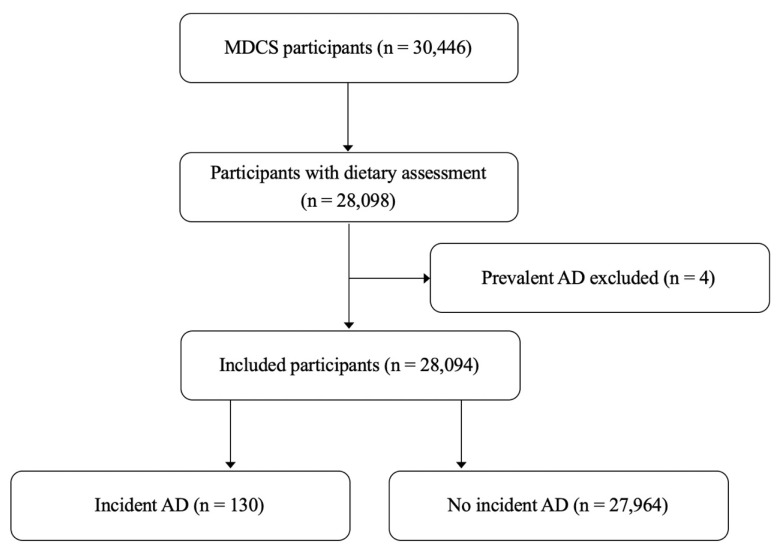
Descriptive flow diagram of study participants with available dietary assessment data and exclusions in the Malmö Diet and Cancer Study (MDCS). AD; aortic dissection.

Men and women aged 46–73 years at baseline were invited to participate in the MDCS. A total of 30,446 participants completed the baseline examination, which included anthropometric measurements, dietary assessment, and a detailed questionnaire on lifestyle and socioeconomic factors. After excluding 2348 individuals with incomplete dietary data and four participants with prevalent AD at baseline, 28,094 participants remained for the final analysis (Figure 1). All participants provided written informed consent prior to inclusion [20].

### 2.2. Lifestyle and Previous Health Condition

Baseline information on participants’ lifestyle and previous health condition characteristics was obtained through self-administered questionnaires, interviews, and physical examinations conducted at the time of recruitment. The lifestyle questionnaire included detailed questions on smoking status, alcohol consumption, physical activity level, educational attainment, medical history, and the use of prescribed medications [18,19]. Clinical measurements such as height, weight, and blood pressure were recorded by trained nurses according to standardized procedures [19,20]. Blood pressure was measured twice in the seated position after ten minutes of rest using a calibrated sphygmomanometer, and body mass index (BMI) was calculated as weight in kilograms divided by height in meters squared (kg/m^2^). Hypertension was defined as a systolic blood pressure ≥ 140 mmHg, diastolic blood pressure ≥ 90 mmHg, or current use of antihypertensive medication, as reported in the questionnaire. Diabetes mellitus was defined as a fasting whole blood glucose concentration ≥ 6.1 mmol/L, self-reported physician-diagnosed diabetes, use of antidiabetic medication, or registration in the local Malmö Diabetes Register [8].

### 2.3. Dietary Assessment and Diet Quality

Dietary habits were assessed using a comprehensive diet history method that combined three components: a seven-day food diary, a 168-item food frequency questionnaire (FFQ), and a one-hour interview with a trained dietician. In the food diary, participants recorded all food items consumed at lunch and dinner, as well as cold beverages. The FFQ documented the frequency and portion size of foods regularly consumed during the past year. A picture booklet was included to assist participants in estimating portion sizes accurately, and during the interview, the dietician clarified information from both the diary and questionnaire, such as typical serving sizes, cooking practices, and recipes [21,22]. Each reported food and beverage were coded according to the Swedish National Food Administration’s nutrient database (Livsmedelsdatabasen), which contains detailed information on the energy and nutrient composition of foods commonly consumed in Sweden. The resulting dietary data were processed using the MDCS’s internally developed dietary calculation system at Lund University, which integrates data from the diary, FFQ, and interview to calculate daily intakes of energy, nutrients, and major food groups [21].

The validity of the combined diet history method was evaluated within the MDCS in comparison with an 18-day weighed food record, considered the reference standard for dietary assessment. The combined method used in the present study demonstrated good agreement and provided more accurate and reliable estimates for large-scale epidemiological research [22].

Diet quality was assessed according to the Swedish nutrition recommendations and dietary guidelines. A diet quality index (DQI) was constructed based on six dietary components: saturated fat, polyunsaturated fat, fish and shellfish, dietary fiber, fruit and vegetables, and sucrose. Participants received one point for each component that met the recommended intake, yielding a total score between zero and six. The specific cut-offs for adherence were polyunsaturated fat 5–10 E%, fish and shellfish ≥ 300 g per week, sucrose ≤ 10 E%, dietary fiber ≥ 2.4 g/MJ, saturated fat ≤ 14 E%, and fruit and vegetables (excluding juice) ≥ 400 g per day. The diet score was further categorized as low (0–1 points), medium (2–4 points), or high (5–6 points), reflecting overall adherence to dietary recommendations [23].

### 2.4. Endpoint Ascertainment

Participants were followed prospectively through national health registers, including the Swedish National Patient Register and the Cause of Death Register until 31 December 2022. These registers contain nationwide data on all hospital admissions and deaths in Sweden, coded according to the International Classification of Diseases (ICD), and together ensure virtually complete ascertainment of both fatal and non-fatal AD events [24,25]. Each participant contributed person-time from baseline until the date of the first registered AD (ICD-10 code I71.0), death, emigration, or end of follow-up, whichever occurred first. Censoring was applied at the time of death, emigration, or the end of the study period, and participants without a registered event by the end of follow-up were treated as censored observations. The median follow-up time was 26.6 years.

AD was identified using ICD-10 code I71.0 (“aortic dissection”) and ICD-9 code 441.0 for events registered before 1997, in accordance with the diagnostic coding transition in the Swedish National Patient Register [8]. Participants with prevalent AD at baseline (*n* = 4) were excluded from all analyses. In total, 130 cases of AD were identified in the MDCS cohort during follow-up through registry linkage.

A validation study was conducted to evaluate the accuracy of the National Patient Register diagnosis of AD. The diagnosis of AD (I71.0) in the Swedish National Patient Register was validated by selecting all cases registered between 1 January 2010 and 31 December 2022. The participants’ hospital records and available radiological images, computed tomography (CT) angiography, were reviewed. In total, 85 participants were identified, and patient records, autopsy protocols, and/or imaging data were available for evaluation of diagnostic accuracy. In total, 64 (75.3%) of the 85 validated cases were confirmed as true AD (Table 1).

### 2.5. Statistical Analyses

Baseline characteristics of participants were summarized as median and interquartile range (IQR) for continuous variables, as well as numbers with corresponding percentages for categorical variables. Comparison between groups were evaluated with chi-square test for nominal data or Mann–Whitney U test for continuous data. Cumulative freedom from incident AD over time was analyzed using the Kaplan–Meier method with Life Tables for men and women, and difference between sexes was evaluated with Log rank test.

Associations between lifestyle exposures and risk of AD were evaluated using Cox proportional hazards regression models, expressed as hazard ratios (HR) with 95% confidence intervals (CIs). The distribution of continuous variables was assessed using the Kolmogorov–Smirnov test. Variables showing skewed distributions (age, BMI, dietary intake variables) were log-transformed prior to analysis and subsequently standardized (z-scored). For these variables, HRs were expressed per one standard deviation (SD) increment to allow comparability across variables. Variables with approximately normal distributions were analyzed on their original scale. Five to ten events per variable in Cox regression were accepted [26]. The proportional hazards assumption was tested by stratifying examined variables. The plots of the estimated log–log survival curves were found to be approximately parallel and fulfilled the proportional hazards assumption.

Three analytical models were used to examine the associations between lifestyle factors and incident AD. Model 1 included adjustment only for age and sex. Model 2 included additional adjustments for major lifestyle and clinical confounders (BMI, hypertension, diabetes, smoking, alcohol intake, educational level, leisure-time physical activity) as well as overall diet quality, providing a fully adjusted estimate. Model 3 evaluated each dietary component separately (saturated fat, polyunsaturated fat, sucrose, fiber, fruits and vegetables, and fish). In this model, each component was analyzed one at a time, while adjusting for the same non-dietary confounders as in Model 2.

For categorical variables, the lowest exposure category was used as the reference group in all analyses, except for alcohol consumption, where the first quintile (low consumption) was chosen as the reference category. This decision was based on evidence from previous large prospective studies showing that zero alcohol consumption is often associated with a higher incidence of cardiovascular disease compared to low or moderate consumption [27,28]. This association may partly reflect the “sick quitter bias”, meaning that individuals who have stopped drinking because of underlying health problems are often classified as lifelong abstainers, which often may lead to an overestimation of disease risk in the non-drinking group [29].

All statistical tests were two-sided, and a *p*-value < 0.05 was considered statistically significant. Statistical analyses were conducted using IBM SPSS Statistics, version 30.0.0.0 (IBM Corp., Armonk, NY, USA).

## 3. Results

### 3.1. Baseline Characteristics

**Figure 2:**
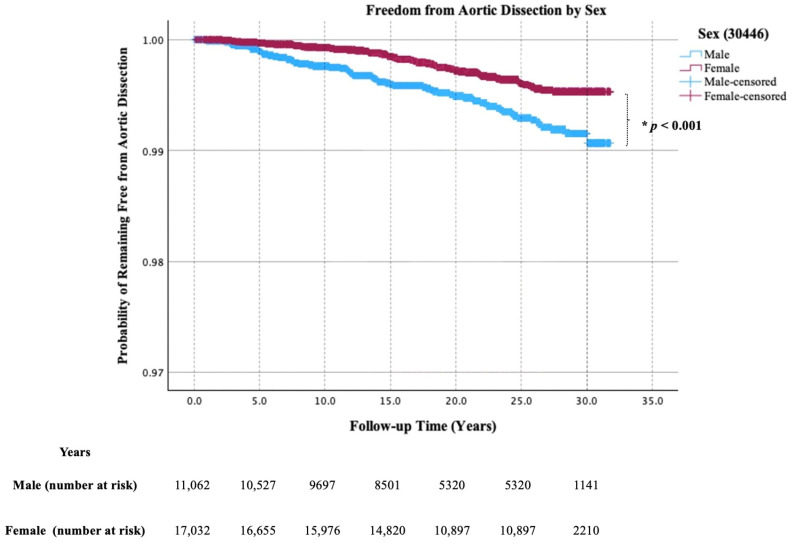
Aortic dissection-free survival by sex. Kaplan–Meier curves comparing men and women in the MDCS cohort. Number at risk is shown below the *X*-axis. * The Log-Rank test was used to compare survival distributions between groups (*p* < 0.001).

During a median follow-up of 26.6 years, 130 (0.5%) incident cases of AD were identified among 28,094 participants in MDCS. At baseline, participants who later developed AD were significantly older than those without incident disease (*p* = 0.001). The Kaplan–Meier analysis showed a higher cumulative incidence of AD in men compared with women (*p* < 0.001, Figure 2). Participants who later developed AD were more frequently hypertensive at baseline compared with those without incident disease (74.4% vs. 61.5%, *p* = 0.003) (Table 2). Higher age (HR 1.76/one standard deviation increment, 95% CI 1.41–2.20), male sex (HR 1.58, 95% CI 1.06–2.34), and hypertension (HR 1.57, 95% CI 1.01–2.44) were each independently associated with increased AD risk (Table 3). Sex-stratified analyses in men (Table S1a,b) and women (Table S2a,b) are shown in supplementary tables. Three out of 67 (4.5%) men and zero out of 63 (0%) women with DM at baseline developed AD (Supplementary Table S1a and Supplementary Table S2a, respectively).

### 3.2. Lifestyle Factors and Aortic Dissection Risk

Current smoking was independently associated with a higher risk of AD compared with never smoking (HR 3.04, 95% CI 1.86–4.98) (Table 3). Zero consumers of alcohol (HR 3.81, 95% CI 1.31–11.03) was independently associated with risk of incident AD in men (Supplementary Table S1b). Leisure-time physical activity was not associated with incident AD (Table 3).

### 3.3. Dietary Factors and Aortic Dissection Risk

Overall diet quality at baseline showed no statistically significant association with incident AD. None of the individual components including saturated and polyunsaturated fat, sucrose, dietary fiber, fruits and vegetables, fish or shellfish showed any significant associations with AD risk (Table 3).

## 4. Discussion

This population-based prospective cohort study with a median follow-up of 26.6 years identified that higher age, male sex, smoking and hypertension were independently associated with an increased risk of AD. In contrast, no significant associations were observed for overall diet quality, specific dietary components, physical activity, diabetes mellitus, BMI, or educational level. High-quality studies investigating the influence of dietary components and physical activity on aortic dissection are indeed scarce. In a recent Mendelian randomization study exploring the causal associations between modifiable risk factors and aortic dissection, hypertension, obesity traits and a diet rich in oily fish, but not physical activity, were shown to have a causal effect on AD [30]. In a retrospective study comparing a sporting and a non-sporting activity group, current smoking was found to facilitate occurrence of AD, especially in the sporting activity group [31]. Taken together, these findings indicate that, unlike atherosclerotic cardiovascular diseases, AD may not be strongly influenced by metabolic or socioeconomic exposures. Instead, the risk might be primarily driven by factors that increase acute aortic wall stress or impair the integrity of the aortic media.

The strong association between hypertension and AD observed in the present study is consistent with previous research. In our cohort, hypertension was present in 74% of participants at the baseline who later developed AD and remained independently associated with incident AD. Similar results were reported in another report using data from MDCS with a median follow-up time of 16 years, where hypertension was present in 86% of AD cases [8]. Likewise, a prospective population-based study identified hypertension as the predominant premorbid condition and noted that poor blood-pressure control before the event was common [32]. Further support comes from large prospective cohorts and meta-analyses showing that the risk of AD increases stepwise with both systolic and diastolic blood pressure, demonstrating a dose-dependent relationship [33], as well as from Mendelian randomization analyses confirming a causal effect of genetically elevated blood pressure, particularly diastolic pressure, on both AD and aortic aneurysm [34]. Hypertension is believed to increase the risk for AD by chronically elevating aortic wall stress, which accelerates medial degeneration and predisposes the aorta to intimal tearing. Sustained high blood pressure leads to fragmentation of elastic fibers, loss of smooth muscle cells, and increased matrix metalloproteinase activity within the aortic media, resulting in structural weakening of the vessel wall [1,2,35].

In the present study, current smoking was independently associated with an increased risk of AD compared with never smoking, even in sex-stratified analyses. Similar findings have been reported in a large population-based cohort, where the risk of aortic mortality increased stepwise with cumulative smoking exposure, reaching almost a fourfold higher risk among those with ≥40 pack-years, demonstrating a dose–response pattern. The study also showed that longer smoking cessation was associated with a steadily decreasing AD risk [36]. Long-term cessation markedly reduced aortic mortality, with risk levels approaching those of never smokers. Consistent with this, evidence from another large community-based cohort with more than two decades of follow-up showed that heavy smoking more than doubled the risk of AD [9]. Smoking is known to exacerbate inflammation and oxidative stress within the aortic wall by promoting vasoconstriction, reducing nitric oxide–mediated vasodilation, and inducing hypoxic injury. Nicotine-driven sympathetic activation further elevates blood pressure, thereby amplifying hemodynamic stress on the aortic wall [37,38]. Together, our findings strengthen the evidence that smoking is an important risk factor for AD and support that smoking cessation should be a part of the primary prevention for AD.

An age-related increase in risk was observed, with each standard deviation in age corresponding to a 76% higher hazard of AD. Consistent with previous population-based studies, the incidence of AD increases markedly with advancing age [5,9,32]. Aging is accompanied by progressive structural and functional alterations in the aortic wall, including fragmentation of elastic fibers, increased collagen cross-linking, and loss of vascular smooth muscle cells, resulting in reduced compliance and increased stiffness. Age-related stiffening of the aortic wall reduces its ability to expand and recoil during systole and diastole, resulting in greater mechanical load on the wall with each cardiac cycle. These changes, particularly when combined with long-standing hypertension, accelerate medial degeneration and impair the wall’s capacity to withstand intraluminal pressure, thereby predisposing to dissection [39].

Among men, zero alcohol consumption was associated with an elevated hazard ratio, consistent with a “sick quitter” effect; this was not observed in women, whose abstainer group may be more heterogeneous, perhaps comprising lifelong abstainers and socially or culturally constrained non-drinkers, and is therefore less influenced by former heavy drinking [40,41].

Leisure-time physical activity showed no significant association with the risk of AD in this cohort. This finding is consistent with results from a large population-based cohort study, which reported no association between walking time or sports participation and mortality from AD [42]. While regular moderate-intensity exercise is well established to improve cardiovascular health and overall quality of life [43], data demonstrating a direct protective effect against dissection are lacking. Despite the well-established cardiovascular benefits of exercise, the absence of an association in this study can be explained by the distinct pathophysiology of AD. Unlike atherosclerotic diseases, in which physical inactivity promotes lipid accumulation and endothelial dysfunction, AD develops mainly because of medial degeneration and acute mechanical wall stress. Therefore, although regular physical activity remains an important component of overall cardiovascular prevention, its direct protective role against AD appears limited.

No significant relationship was observed between overall diet quality, specific dietary components, and the risk of AD. The lack of association in the present study may have two explanations. First, dietary exposures are primarily related to atherosclerotic and metabolic pathways, which are less relevant to the medial degeneration underlying AD. The specific dietary components included in the index, such as intake of saturated fat, fiber, and fruit and vegetables, are known to influence lipid metabolism and endothelial function [44,45,46,47] but may have limited effects on the structural integrity of the aortic media. Second, the relatively low number of participants with incident AD limits statistical power, increasing the risk of Type II error and reducing the ability to detect weaker associations.

AD appears primarily to be a disease of acute structural failure, driven by hemodynamic stress acting on a structurally weakened aortic media. Approximately 58% of dissections occur in the ascending aorta, 42% in the descending thoracic aorta, and only about 1–2% below the diaphragm [48,49]. This distribution reflects the structural and biomechanical gradients along the aorta. The thoracic aorta, particularly the ascending segment, is rich in elastin and relatively poor in collagen, providing high compliance but reduced structural integrity. This composition enables the aorta to absorb the high ejection pressure from the left ventricle and maintain continuous blood flow during diastole through the Windkessel effect, but it also predisposes the wall to layer separation under sustained or excessive shear stress [50,51]. Chronic hypertension, aging, smoking, and bicuspid aortic valve further elevate wall stress and accelerate medial degeneration, characterized by elastin fragmentation, smooth muscle cell loss, and proteoglycan accumulation. These degenerative changes weaken the media, increasing vulnerability to intimal tearing and propagation of a dissection flap, particularly in the ascending and proximal descending aorta [4,52]. In contrast, aortic aneurysm represents a chronic degenerative process that develops gradually over time, and its anatomic distribution differs markedly from AD. Whereas dissections predominantly involve the thoracic aorta, 60–70% of aneurysms occur in the infrarenal abdominal aorta, with only 30% in the ascending aorta and 10% in the descending thoracic aorta [1]. This contrasting distribution reflects regional differences in wall structure and flow dynamics. The abdominal aorta, with fewer elastic lamellae, more collagen, and limited vasa vasorum perfusion, is stiffer and less able to buffer pulsatile flow. These features, together with low and oscillatory shear stress, make the abdominal segment particularly susceptible to injury in the presence of cardiovascular risk factors such as hypertension, smoking, aging, and dyslipidemia. These factors promote chronic inflammation, matrix degradation, and impaired endothelial function, mechanisms central to aneurysm formation but less directly involved in the acute mechanical failure that characterizes AD.

Strengths of this study include its large population-based cohort and the long follow-up period exceeding 26 years. Another important strength was the validation of registry-based diagnoses, which confirmed true AD in 75% of reviewed cases. Although the validation itself is a strength, the positive predictive value of 75% shows that a notable proportion of registry-identified cases were misclassified, which constitutes an important limitation. Additional limitations were that lifestyle and dietary exposures were recorded only once at baseline. If people changed their habits during follow-up, their baseline data would no longer be accurate, making it harder to see true relationships between lifestyle factors and AD. The study relies on self-reported lifestyle data, which may introduce recall bias or underreporting, particularly for alcohol intake, dietary components, and smoking history. Despite the large cohort size, the absolute number of AD cases was relatively low, limiting statistical power and reducing the ability to detect weaker associations. Residual confounding—i.e., factors that were not measured such as triggers—for aortic tear in susceptible individuals with connective tissue disorders, aortic aneurysm or dilatation, bicuspid aortic valve-related aortopathy, heavy lifting, stimulant use, trauma, pregnancy/postpartum states, and cardiovascular procedures, or not measured with perfect accuracy, such as genetic predisposition, long-term blood pressure control, or adherence to medications, may still have affected the risk estimates. Together, these limitations mean that small or modest associations may not have been detectable in this study. This cohort of middle-aged participants were predominantly Swedish, and the findings may not equally apply to younger populations and ethnic minorities.

## 5. Conclusions

In summary, age, male sex, current smoking, and hypertension, rather than diet and physical activity, were associated with risk of AD in this prospective population-based study. These results support the recommendation of early detection and strict management of elevated blood pressure and smoking cessation as key strategies to reduce the incidence of this life-threatening condition.

## Figures and Tables

**Table 1:** Validation of participant data registered as I71.0 (Aortic dissection).

	Number of Participants	In-Hospital Mortality
Total number of participants	85	
		
Diagnosis confirmed (%)	64 (75.3)	20 (31.3)
CT angiography (%)	51 (79.7)	
Autopsy (%)	13 (20.3)	
Hypertension (%)	55/61 (90.2)	
Acute dissection (%)	52 (81.3)	
Type Stanford A, DeBakey I (%)	22 (34.4)	8 (36.4)
Type Stanford A, DeBakey II (%)	20 (31.2)	11 (55.0)
Type Stanford B, DeBakey III (%)	20 (31.2)	1 (5.0)
Infrarenal aortic dissection	2 (3.1)	0 (0)
Complicated * (%)	31 (48.4)	17 (54.8)
Rupture or hemopericardium (%)	24 (37.5)	15 (62.5)
Organ ischemia (%)	8 (12.5)	3 (37.5)
Acute operation (%) ^#^	11 (17.2)	0 (0)
		
Diagnosis confirmed not to be aortic dissection (%)	19 (22.4)	
Aorta ascendens ectasia (%)	5	
Mycotic aortic arch aneurysm (%)	1	
Thoracic aortic aneurysm (%)	1	
Thoracoabdominal aortic aneurysm (%)	1	
Ruptured abdominal aortic aneurysm (%)	1	
Ruptured plaque in arcus aorta (%)	1	
Penetrating ulcer in the thoracic aorta (%)	2	
Para-aortic hematoma in abdominal aorta (%)	1	
Ruptured iliac aneurysm (%)	1	
Iatrogenic dissection of descending aorta (%)	1	
Floating thrombus in thoracic aorta (%)	1	
Rupture of left chamber with acute myocardial infarction and hemopericardium (%)	1	
Muscular dystrophy (totally miscoded)	1	
Clinical suspicion of acute aortic dissection before CT angiography where CT showed no aortic dissection (%)	1	
		
Diagnosis not confirmed (%)	2 (2.4)	
Unclear diagnosis (no autopsy) (%)	2	

* Complicated (shock, rupture or organ ischemia), ^#^ nine undergoing open thoracic surgery, CT: Computed tomography.

**Table 2:** Baseline characteristics of 28,094 study participants with and without incident aortic dissection in the MDCS cohort.


Characteristics	Incident AD (*n* = 130)	No Incident AD (*n* = 27,964)
Demographics and previous health conditions		
Male sex (%)	67 (51.5)	10,995 (39.43)
Age (years)	60.8 (54.1–65.8)	57.8 (51.3–64.2)
BMI (kg/m^2^)	24.8 (22.6–27.7)	25.3 (23.0–28.0) (*n* = 27,922)
Hypertension (%)	96/129 (74.4)	17,142/27,894 (61.5)
Diabetes mellitus (%)	3 (2.3)	1227 (4.4)
Lifestyle factors		
Alcohol Consumption (%)		
Zero consumers	12 (9.2)	1794 (6.4)
Quintile 1 (<0.9 g/day for women/ <3.4 g/day for men)	19 (14.6)	5236 (18.7)
Quintile 2 (0.9–4.3 g/day for women/3.4–9.1 g/day for men)	22 (16.9)	5238 (18.7)
Quintile 3 (4.4–8.1 g/day for women/9.2–15.7 g/day for men)	25 (19.2)	5237 (18.7)
Quintile 4 (8.2–14.0 g/day for women/15.7–25.7 g/day for men)	26 (20.0)	5228 (18.7)
Quintile 5 (>14.0 g/day for women/ >25.7 g/day for men)	26 (20.0)	5231 (18.7)
Smoking (%)		
Never	34 (26.2%)	10,610 (38.0%)
Former	46 (35.4%)	9458 (33.8%)
Current	50 (38.5%)	7884 (28.2%)
Leisure Time Physical Activity (%)		
<7.5 MET-h/week	9/129 (7.0)	2722/27,762 (9.8)
7.5–15.0 MET-h/week	21/129 (16.3)	4139/27,762 (14.9)
15.1–25.0 MET-h/week	29/129 (22.5)	6380/27,762 (23.0)
25.1–50.0 MET-h/week	50/129 (38.8)	10,067/27,762 (36.3)
>50.0 MET-h/week	20/129 (15.5)	4454/27,762 (16.0)
Educational Level (%)		
Less than 9 years	52 (40.0)	11,724/27,893 (42.0)
Elementary school (9–10 years)	32 (24.6)	7297/27,893 (26.2)
Elementary + upper secondary school (9–13 years)	19 (8.0)	2472/27,893 (8.9)
University studies, no degree	11 (14.6)	2431/27,893 (8.7)
University studies, with degree	16 (12.3)	3969/27,893 (14.2)
Diet Quality		
Low (%)	19 (14.6)	4275 (15.3)
Medium (%)	95 (73.1)	19,935 (71.3)
High (%)	16 (12.3)	3754 (13.4)
Diet score (0–6)	3 (2–3)	3 (2–4)
Dietary Components		
Total energy intake (kcal/day)	2287.2 (1978.8–2715.9)	2184.6 (1817.5–2631.9)
Saturated fat (E%)	16.2 (13.7–18.7)	15.7 (13.6–18.3)
Polyunsaturated fat (E%)	5.7 (4.8–6.6)	5.8 (4.8–6.8)
Sucrose (E%)	8.4 (6.5–11.2)	8.0 (6.1–10.3)
Fiber (g/MJ)	2.0 (1.7–2.4)	2.1 (1.8–2.6)
Fruit and vegetables (g/day)	336.8 (245.7–420.6)	346.4 (245.3–474.2)
Fish (g/week)	294.6 (204.3–470.9)	277.1 (150.0–440.3)

Data are *n* (%) or median (interquartile range, IQR). MDCS; Malmö Diet and Cancer Study, BMI; body mass index, E; energy, MET; metabolic equivalent of task, MJ; megajoule.

**Table 3:** Age-, sex-, and multivariable-adjusted associations between lifestyle factors and risk of aortic dissection among 28,094 study participants.


Characteristics	Age-and Sex Adjusted HR (95% CI)	Multivariable * Adjusted HR (95% CI)
Demographics and previous health conditions		
Male sex (%)	1.69 (1.16–2.46)	1.58 (1.06–2.34)
Age (years)	1.66 ^a^ (1.35–2.04)	1.76 ^a^ (1.41–2.20)
BMI (kg/m^2^)	0.92 ^a^ (0.75–1.13)	0.93 ^a^ (0.76–1.15)
Hypertension (%)	1.48 (0.96–2.29)	1.57 (1.01–2.44)
Diabetes mellitus (%)	0.70 (0.22–2.22)	0.73 (0.23–2.33)
Lifestyle factors		
Alcohol Consumption (%)		
Zero consumers	1.93 (0.91–4.09)	1.95 (0.89–4.25)
Quintile 1 (<0.9 g/day for women/ <3.4 g/day for men)	1 (Ref)	1 (Ref)
Quintile 2 (0.9–4.3 g/day for women/3.4–9.1 g/day for men)	0.88 (0.45–1.71)	0.89 (0.46–1.72)
Quintile 3 (4.4–8.1 g/day for women/9.2–15.7 g/day for men)	1.10 (0.59–2.07)	1.07 (0.57–2.01)
Quintile 4 (8.2–14.0 g/day for women/15.7–25.7 g/day for men)	1.19 (0.64–2.22)	1.08 (0.57–2.03)
Quintile 5 (>14.0 g/day for women/ >25.7 g/day for men)	1.27 (0.68–2.40)	1.05 (0.55–2.01)
Smoking%		
Never	1 (Ref)	1 (Ref)
Former	1.50 (0.91–2.46)	1.59 (0.96–2.64)
Current	2.85 (1.77–4.61)	3.04 (1.86–4.98)
Leisure Time Physical Activity (%)		
<7.5 MET-h/week	1 (Ref)	1 (Ref)
7.5–15.0 MET-h/week	1.47 (0.61–3.54)	1.48 (0.61–3.57)
15.1–25.0 MET-h/week	1.51 (0.66–3.46)	1.56 (0.68–3.58)
25.1–50.0 MET-h/week	1.33 (0.60–2.98)	1.40 (0.62–3.16)
>50.0 MET-h/week	1.21 (0.50–2.92)	1.28 (0.52–3.10)
Educational Level (%)		
Less than 9 years	1 (Ref)	1 (Ref)
Elementary school (9–10 years)	1.11 (0.69–1.78)	1.19 (0.73–1.93)
Elementary + upper secondary school (9–13 years)	1.69 (0.93–3.05)	1.84 (1.01–3.36)
University studies, no degree	0.93 (0.44–1.98)	1.01 (0.47–2.16)
University studies, with degree	1.09 (0.60–2.01)	1.24 (0.67–2.32)
Diet Quality		
	Model 1	Model 2
Low (%)	1 (Ref)	1 (Ref)
Medium (%)	1.03 (0.60–1.76)	1.12 (0.65–1.92)
High (%)	0.78 (0.37–1.64)	0.84 (0.39–1.84)
Diet score (0–6)	0.97 (0.80–1.18)/ point increase	0.99 (0.81–1.21)/point increase
Dietary Components		
	Model 1	Model 3
Total energy intake (kcal/day)	1.07 ^a^ (0.86–1.32)	1.03 ^a^ (0.83–1.29)
Saturated fat (E%)	1.06 ^a^ (0.87–1.28)	1.01 ^a^ (0.83–1.22)
Polyunsaturated fat (E%)	0.95 ^a^ (0.79–1.16)	0.93 ^a^ (0.77–1.13)
Sucrose (E%)	1.10 ^a^ (0.90–1.34)	1.09 ^a^ (0.89–1.33)
Fiber (g/MJ)	0.84 ^a^ (0.69–1.02)	0.97 ^a^ (0.80–1.18)
Fruit and vegetables (g/day)	0.92 ^a^ (0.76–1.11)	0.95 ^a^ (0.78–1.16)
Fish (g/week)	1.04 ^a^ (0.85–1.27)	1.04 ^a^ (0.85–1.27)

MDCS; Malmö Diet and Cancer Study, HR; hazard ratio, BMI; body mass index, E; energy, MET; metabolic equivalent of task, MJ; megajoule. ^a^ HRs are expressed per 1 standard deviation increment. * Multivariable models include all risk factors and diet quality respective dietary component variables. Dietary component variables were not mutually adjusted nor with diet quality.

## Data Availability

The datasets analyzed during the current study are not publicly available due to the nature of sensitive personal data and study materials. However, procedures for sharing data, analytical methods, and study materials for reproducing the results following Swedish legislation can be arranged by contacting the corresponding author or study organization (https://www.malmo-kohorter.lu.se/malmo-kost-cancer-mkc, accessed on 27 December 2024).

## Supplementary Materials

The following supporting information can be downloaded at https://www.mdpi.com/article/10.3390/jcdd13030142/s1. Table S1a. Baseline characteristics of 11,062 male participants with and without incident aortic dissection in the MDCS cohort; Table S1b. Age- and sex-adjusted and multivariable-adjusted associations between lifestyle factors and risk of aortic dissection among 11,062 male participants. Table S2a. Baseline characteristics of 17,032 female participants with and without incident aortic dissection in the MDCS cohort; Table S2b. Age- and sex-adjusted and multivariable-adjusted associations between lifestyle factors and risk of aortic dissection among 17,032 female participants.
