# Supplementary material for: Association Between Diet Quality, Physical Activity, and the Risk of Aortic Dissection—A Prospective Cohort Study
Source: J Cardiovasc Dev Dis. 2026 Mar 18;13(3):142. doi: 10.3390/jcdd13030142 (PMC13026820; doi:10.3390/jcdd13030142)
Supplement: Supplementary file 1 [file jcdd-13-00142-s001.zip › jcdd-4160980-supplementary.pdf]

Table S1a. Baseline characteristics of 11,062 male participants with and without incident aortic dissection in the MDCS cohort.

Characteristics	Incident AD (n = 130)	No Incident AD (n = 27,964)
**Demographics and previous health condition**		
Age (years)	60.77 (53.24–65.98)	59.12 (53.04–64.71)
Hypertension (%)	50/66 (75.8)	7,521/10,964 (68.6)
**Alcohol Consumption (%)**		
Zero consumers	7 (10.4)	489 (4.4)
Quintile 1 (<0.9 g/day for women/ <3.4 g/day for men)	10 (14.9)	2,098 (19.1)
Quintile 2 (0.9–4.3 g/day for women/3.4–9.1 g/day for men)	12 (17.9)	2,106 (19.2)
Quintile 3 (4.4–8.1 g/day for women/9.2–15.7 g/day for men)	11 (16.4)	2,108 (19.2)
Quintile 4 (8.2–14.0 g/day for women/15.7–25.7 g/day for men)	14 (20.9)	2,097 (19.1)
Quintile 5 (>14.0 g/day for women/ >25.7 g/day for men)	13 (19.4)	2,097 (19.1)
**Smoking%**		
Never	14 (20.9)	3,134/10,989 (28.2)
Former	28 (41.8)	4,752/10,989 (43.2)
Current	25 (37.3)	3,134/10,989 (28.2)
**Leisure Time Physical Activity (%)**		
<7.5 MET-h/week	5/67 (7.5)	1,120/10,914 (9.8)
7.5–15.0 MET-h/week	13/67 (16.3)	1,608/10,914 (14.9)
15.1–25.0 MET-h/week	15/67 (22.5)	2,406/10,914 (23.0)
25.1–50.0 MET-h/week	24/67 (38.8)	3,811/10,914 (36.3)
>50.0 MET-h/week	10/67 (15.5)	1,969/10,914 (16.0)
**Diet Quality**		
Diet score (0–6)	3 (2–3)	3 (2–4)

Data are n (%) or median (interquartile range, IQR). MDCS; Malmö Diet and Cancer Study, MET; metabolic equivalent of task.

Table S1b. Age- and sex-adjusted and multivariable-adjusted associations between lifestyle factors and risk of aortic dissection among 11,062 male participants.

Characteristics	Multivariable * Adjusted HR (95% CI)
**Demographics and previous health condition**	
Age (years)	1.57 [a] (1.13–2.19)
Hypertension (%)	1.64 (0.85–3.16)
**Alcohol Consumption (%)**	
Zero consumers	3.81 (1.31–11.03)
Quintile 1 (<0.9 g/day for women/ <3.4 g/day for men)	1 (Ref)
Quintile 2 (0.9–4.3 g/day for women/ 3.4–9.1 g/day for men)	1.03 (0.4–2.66)
Quintile 3 (4.4–8.1 g/day for women/ 9.2–15.7 g/day for men)	0.88 (0.33–2.34)
Quintile 4 (8.2–14.0 g/day for women/ 15.7–25.7 g/day for men)	1.1 (0.42–2.69)
Quintile 5 (>14.0 g/day for women/ >25.7 g/day for men)	1.19 (0.48–2.99)
**Smoking%**	
Never	1 (Ref)
Former	1.54 (0.72–3.3)
Current	2.99 (1.38–6.49)
**Leisure Time Physical Activity (%)**	
<7.5 MET-h/week	1 (Ref)
7.5–15.0 MET-h/week	1.62 (0.51–5.17)
15.1–25.0 MET-h/week	1.29 (0.41–4.01)
25.1–50.0 MET-h/week	1.3 (0.44–3.87)
>50.0 MET-h/week	0.9 (0.26–3.12)
**Diet Quality**	

Diet score (0–6)	0.99 [a] (0.75–1.32)/point increase

MDCS; Malmö Diet and Cancer Study, HR; hazard ratio, MET; metabolic equivalent of task. [a] HRs are expressed per 1 standard deviation increment. All variables entered as covariates into the multivariable model.

Table S2a. Baseline characteristics of 17,032 female participants with and without incident aortic dissection in the MDCS cohort.

Characteristics	Incident AD (n = 130)	No Incident AD (n = 27,964)
**Demographics and previous health condition**		
Age (years)	61.4 (54.98–65.22)	56.67 (50.0–63.77)
Hypertension (%)	46/63 (73)	9,621/16,930 (56.8)
**Alcohol Consumption (%)**		
Zero consumers	5 (7.9)	1,305 (7.7)
Quintile 1 (<0.9 g/day for women/ <3.4 g/day for men)	9 (14.3)	3,138 (18.5)
Quintile 2 (0.9–4.3 g/day for women/3.4–9.1 g/day for men)	10 (15.9)	3,132 (18.5)
Quintile 3 (4.4–8.1 g/day for women/9.2–15.7 g/day for men)	14 (22.2)	3,129 (18.4)
Quintile 4 (8.2–14.0 g/day for women/15.7–25.7 g/day for men)	12 (19.0)	3,131 (18.5)
Quintile 5 (>14.0 g/day for women/ >25.7 g/day for men)	13 (20.6)	3,134 (18.5)
**Smoking%**		
Never	20 (31.7)	7,507/16,963 (44.3)
Former	18 (28.6)	4,706/16,963 (27.7)
Current	25 (39.7)	4,750/16,963 (28.2)
**Leisure Time Physical Activity (%)**		
<7.5 MET-h/week	4/62 (6.5)	1,602/16,848 (9.5)
7.5–15.0 MET-h/week	8/62 (12.9)	2,531/16,848 (15.0)
15.1–25.0 MET-h/week	14/62 (22.6)	3,974/16,848 (23.6)
25.1–50.0 MET-h/week	26/62 (41.9)	6,256/16,848 (37.1)
>50.0 MET-h/week	10/62 (16.1)	2,485/16,848 (14.7)
**Diet Quality**		

Diet score (0–6)	3 (2–4)	3 (2–4)

Data are n (%) or median (interquartile range, IQR). MDCS; Malmö Diet and Cancer Study, MET; metabolic equivalent of task.

Table S2b. Age- and sex-adjusted and multivariable-adjusted associations between lifestyle factors and risk of aortic dissection among 17,032 female participants.

Characteristics	Multivariable * Adjusted HR (95% CI)
**Demographics and previous health condition**	
Age (years)	1.99 [a] (1.46–2.73)
Hypertension (%)	1.67 (0.88–3.19)
**Alcohol Consumption (%)**	
Zero consumers	0.57 (0.12–2.66)
Quintile 1 (<0.9 g/day for women/ <3.4 g/day for men)	1 (Ref)
Quintile 2 (0.9–4.3 g/day for women/ 3.4–9.1 g/day for men)	0.64 (0.23–1.79)
Quintile 3 (4.4–8.1 g/day for women/ 9.2–15.7 g/day for men)	1.33 (0.56–3.15)
Quintile 4 (8.2–14.0 g/day for women/ 15.7–25.7 g/day for men)	1.32 (0.55–3.15)
Quintile 5 (>14.0 g/day for women/ >25.7 g/day for men)	1.15 (0.46–2.85)
**Smoking%**	
Never	1 (Ref)
Former	1.53 (0.75–3.13)
Current	3.11 (1.59–6.1)
**Leisure Time Physical Activity (%)**	
<7.5 MET-h/week	1 (Ref)
7.5–15.0 MET-h/week	4.21 (0.52–34.26)
15.1–25.0 MET-h/week	4.65 (0.61–35.6)
25.1–50.0 MET-h/week	4.84 (0.65–36.12)
>50.0 MET-h/week	5.1 (0.64–40.13)
**Diet Quality**	

Diet score (0–6)	0.99 [a] (0.75–1.3)/point increase

MDCS; Malmö Diet and Cancer Study, HR; hazard ratio, MET; metabolic equivalent of task. [a] HRs are expressed per 1 standard deviation increment. All variables entered as covariates into the multivariable model.
